# Oral Intake of *Streptococcus thermophilus* iHA318 Mitigates Dry Eye Symptoms in a Randomized Clinical Study

**DOI:** 10.3390/biomedicines13040931

**Published:** 2025-04-10

**Authors:** Chieh-Hung Yen, Yu-Wei Chang, Yen-Ling Sun, Yi-Yun Hung, Wei-Chieh Liao, Tsung-Han Lu, Pin-Chao Huang, Han-Hsin Chang, Meei-Yn Lin, David Pei-Cheng Lin

**Affiliations:** 1 Department of Ophthalmology, Jen-Ai Hospital, Taichung 412224, Taiwan; chiehhungyen@gmail.com; 2 Department of Ophthalmology, Chang Gung Memorial Hospital at Linkou, Taoyuan 333423, Taiwan; 3 Graduate Institute of Biomedical Engineering, Chang Gung University, Taoyuan 333323, Taiwan; 4 College of Medicine, Chang Gung University, Taoyuan 333323, Taiwan; 5 Department of Medical Laboratory and Biotechnology, Chung Shan Medical University, Taichung 402306, Taiwan; golden3p@csmu.edu.tw (Y.-W.C.); lilliansun0429@gmail.com (Y.-L.S.); jennyhung0820@gmail.com (Y.-Y.H.); a0966150629@gmail.com (W.-C.L.); j5657849044@gmail.com (P.-C.H.); 6 Department of Clinical Laboratory, Chung Shan Medical University Hospital, Taichung 402306, Taiwan; 7 Institute of Medicine, Chung Shan Medical University, Taichung 402306, Taiwan; rightlu1114@gmail.com; 8 Department of Nutrition, Chung Shan Medical University, Taichung 402306, Taiwan; jhhc@csmu.edu.tw; 9 Department of Food Science and Biotechnology, National Chung Hsing University, Taichung 402202, Taiwan; 10 Department of Ophthalmology, Chung Shan Medical University Hospital, Taichung 402306, Taiwan

**Keywords:** dry eye symptoms, probiotics, *Streptococcus thermophilus* iHA318, sialic acid, NLRP3 inflammasome

## Abstract

**Background/Objectives**: A probiotic *Streptococcus thermophilus* (iHA318) has been demonstrated to alleviate dry eye symptoms in a mouse model. This study investigated its effects on dry eye mitigation in a clinical trial. **Methods**: A total of 68 volunteers were recruited in the double-blind clinical trial and randomly divided into a probiotic group and a placebo group. The probiotic group received iHA318 capsules daily for 35 days via oral intake, while the placebo group received microcrystalline cellulose capsules. Assessments before and after the intervention were performed for the tear volume (TV), tear break-up time (TBUT), tear osmolarity (Osmo), serum sialic acid (SA) concentrations, and the Ocular Surface Disease Index (OSDI), and an impression cytology analysis was conducted for immunofluorescence detection of NLRP3 expression. **Results**: The tear volume was significantly increased in the probiotic group, although a placebo effect was observed in the placebo group. The probiotic group showed a significant reduction in tear osmolarity, an extended TBUT, and an improved OSDI score. These parameters were also observed in the placebo group without statistical significance. In addition, the serum SA was significantly increased in the probiotic group in contrast to a slight non-significant increase in the placebo group. Reductions in NLRP3 inflammasome activation and OSDI were found only in the probiotic group. **Conclusions**: In conclusion, a significant improvement in major dry eye symptoms after iHA318 treatment was observed compared to the placebo group.

## 1. Introduction

Dry eye syndrome (DES) is a chronic multifactorial disease characterized by inadequate tear quality or quantity to lubricate the ocular surface properly [1]. Its prevalence is notably higher among the elderly, women, and individuals with systemic comorbidities or environmental exposure [2]. Although particularly common in Asian populations, DES is a growing concern worldwide [3,4]. DES is influenced by multiple demographic and biological factors. Aging is associated with reduced tear production, meibomian gland dysfunction, and ocular surface sensitivity [5]. Gender differences, particularly the higher prevalence of DES in women, are linked to hormonal fluctuations, including reduced androgen and estrogen levels, which affect lacrimal gland function and mucin secretion [6]. Systemic medications (e.g., antihistamines and antidepressants), environmental exposure (e.g., screen use and air pollution), and systemic conditions like diabetes or autoimmune diseases further exacerbate ocular surface instability [7]. Additionally, recent developments such as the COVID-19 pandemic have contributed to increased DES burden through prolonged digital screen exposure, altered lifestyle patterns, and reduced blink rates [8,9]. Furthermore, extensive use of face masks—especially in clinical and public settings—has been linked to mask-associated dry eye (MADE), likely due to redirected airflow causing tear film instability [10]. These emerging challenges highlight the urgent need for alternative DES management strategies with favorable safety profiles. Currently, artificial tears and anti-inflammatory agents are commonly used as long-term therapies for patients with DES. However, chronic applications of these medications can cause some adverse effects. For example, long-term use of topical corticosteroids is known to cause ocular hyperemia, ocular hypertension, corneal calcification, and liability to infection [11]. Similarly, although artificial tears are considered safe and are commonly used for symptomatic relief, they have notable limitations, including a short retention time, a lack of anti-inflammatory effect, and potential epithelial toxicity from preservatives (e.g., benzalkonium chloride) with chronic use [12]. Thus, an unmet need exists to develop effective therapeutic interventions with favorable safety profiles for DES patients.

The microbiome has been known to exert immunomodulation, involving both health maintenance and the pathogenesis of diseases [13,14]. Several studies showed that the dysregulation of the microbiome plays an important role in chronic inflammatory disorders, such as in the status of DES [15]. Yun et al. showed that oral gavage of probiotics could improve dry eye syndrome in an animal model by modulating the inflammatory response and gut microbiota composition [16]. Lee et al. reported that the oral administration of Lactobacillus fermentum HY7302 improved dry eye symptoms in a mouse model [17]. Wang et al. observed that dysbiosis modulates ocular surface inflammatory response to liposaccharide [18], leading to a proposal of a gut–eye–lacrimal gland–microbiome axis in Sjögren syndrome by Trujillo-Vargas et al. [19]. This hypothesis of an ocular surface effect under the influence of the gut microbiome was supported by Schaefer et al., who demonstrated that gut microbiota from Sjögren syndrome patients caused a reduction in T regulatory cells in the lymphoid organs and aggravated cornea barrier disruption in a desiccation-induced dry eye model [20].

Although microbiota-based immunomodulation has been proposed for DES therapy, previous studies have thus far provided limited evidence of its efficacy. Heydari et al. studied the effect of *Latilactobacillus sakei* formulations on the clinical and immunological outcomes of dry eye patients [21]. They found that the oral intake of the probiotic capsule did not affect the patients. Therefore, evidence of using oral probiotics for DES mitigation in clinical settings remains elusive and demands more investigations. We previously reported that the oral intake of a specific probiotic, *Streptococcus thermophilus* iHA318, improved dry eye symptoms by mitigating ocular surface damage in a mouse model [22]. To extend previous investigations, this study investigated the effects of oral *Streptococcus thermophilus* iHA318 intakes for dry alleviation in a clinical setting. The focus was on clinically assessed DES signs and symptoms, including TV, tear osmolarity, TBUT, and OSDI, and on the serum SA levels. Additionally, an impression cytology analysis was conducted for NLRP3 expression detection on the ocular surface to further elucidate the underlying mechanisms implicated in the gut microbiota–ocular surface axis.

## 2. Materials and Methods

### 2.1. Participant Enrollment

**Figure 1:**
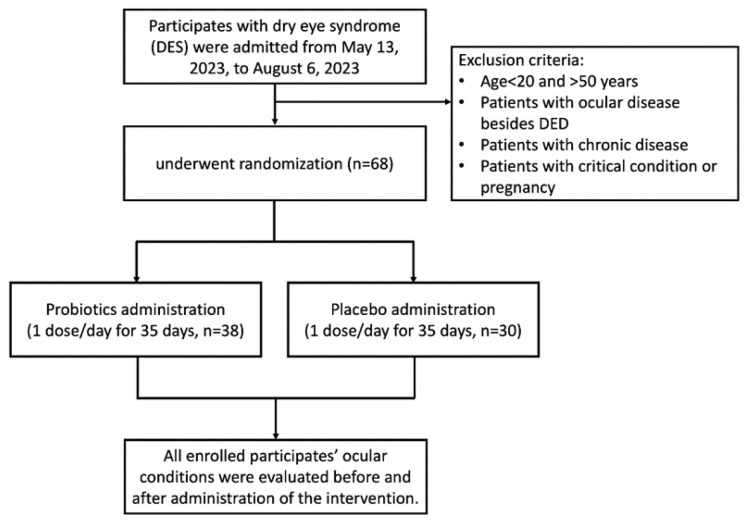
Flowchart of enrollment of 68 DES participants in clinical trial.

The protocol of this clinical trial was approved by the Institutional Review Board (IRB) of Jen Ai Hospital (Taichung, Taiwan; IRB Number: 2302180001, followed by registration with a ClinicalTrials.gov ID: NCT05906381; 15 June 2023) and conducted with informed consent obtained from all participants. All methods were performed following the relevant guidelines and regulations. All participants were examined by an ophthalmologist during the screening process. Those who had been diagnosed with dry eye disease (DED), autoimmune diseases (e.g., Sjögren’s syndrome), ocular surface diseases other than DES, or chronic systemic illnesses or had used steroid-based or antibiotic eye treatments (such as corticosteroids or cyclosporine) were excluded from participation. Only participants with DES but without a confirmed DED diagnosis were enrolled. While over-the-counter artificial tear drop use was not an exclusion criterion, all participants confirmed no use of prescription dry eye therapies. The aim of this was to minimize prior treatment effects on study outcomes. Participants aged 20–65 years presenting with dry eye symptoms, including low tear volume (Schirmer’s test < 10 mm) or an OSDI score greater than 25, and who did not meet any exclusion criteria, were enrolled in the human clinical trial (Figure 1). Patients aged less than 20 years or more than 50 years with ocular diseases besides dry eye disease (DED), chronic diseases, critical conditions, or who were pregnant were excluded from this study. The enrolled participants were randomly divided into a probiotic group and a placebo group for double-blind study. All participants were instructed not to alter their usual dietary habits or lifestyle patterns during the intervention to minimize potential confounding factors. All participants were evaluated immediately on enrollment and on day 36 after a 35-day trial period.

### 2.2. Administration of iHA318 Probiotics and Placebo Capsules

All enrolled participants received either a probiotic capsule or a placebo capsule per day for 35 days. The 35-day intervention period was selected based on previous preclinical evidence demonstrating measurable effects of iHA318 within 4–5 weeks, as well as clinical studies such as a report published by Tavakoli et al. [23], which reported significant improvement in OSDI scores after 1 month of probiotic supplementation in dry eye patients. This timeframe was thus considered appropriate for assessing early-stage symptomatic relief [23]. The components are listed in Table 1, which were prepared by NUTRAREX BIOTECH company (Taichung, Taiwan).

### 2.3. Tear Volume Measurement (Schirmer’s Test)

Tear secretion was evaluated using the Schirmer II test following standardized procedures described in prior studies [24]. One drop of 0.5% proparacaine hydrochloride was instilled into the lower conjunctival sac to eliminate reflex tearing. After a few minutes, a sterile Schirmer strip (Haag-Streit, Bishop’s Stortford, UK) was placed in the lower eyelid of each eye at the junction of the middle and lateral third. Participants were instructed to keep their eyes gently closed, and the length of moistened strip (in millimeters) was recorded after 5 min to determine basal tear production.

### 2.4. Ocular Surface Disease Index (OSDI) Evaluation

The OSDI is a validated questionnaire commonly used to assess the severity of symptoms related to ocular surface diseases, such as DES [25]. It comprises a series of questions (12 items) related to ocular discomfort, visual disturbances, and the impact of these symptoms on daily activities. Participants are asked to rate the frequency and severity of their symptoms over the past week on a scale ranging from 0 to 100, with higher scores indicating more severe symptoms and more significant impairment of vision-related function. A formula was used to determine the final OSDI value: OSDI = the sum of scores × 25/number of questions answered.

### 2.5. Tear Break-Up Time (TBUT) Test

TBUT measurement represents a quick and easy method to evaluate tear film stability, serving as a standard diagnostic procedure in dry eye clinics [26]. In the present study, TBUT was assessed using sodium fluorescein dye (FLUO 900, Haag-Streit, Bishop’s Stortford, UK) prepared in 0.9% NaCl saline. One drop (~5 μL) of prepared fluorescein dye solution was gently applied to the inferior palpebral conjunctiva of each eye. Participants were asked to blink several times to distribute the dye evenly across the ocular surface. TBUT was measured as the time (in seconds) between the last complete blink and the first appearance of a dry spot, observed under cobalt blue illumination using a slit lamp (Topcon SL-2E, Topcon, Tokyo, Japan).

### 2.6. Tear Osmolarity Measurement

Tear osmolarity was measured before and after the administration of iHA318 or placebo. The measurement was performed by using the TearLab™ osmolarity system (TearLab™ Corp., San Diego, CA, USA) according to the manufacturer’s instructions.

### 2.7. Enzyme-Linked Immunosorbent Assay (ELISA)

Serum samples were obtained from the enrolled participants in the clinical trial. The sialic acid levels were measured using ELISA kits (Cat# E-BC-K068-M-500, Elabscience, Houston, TX, USA) following the manufacturers’ instructions.

### 2.8. Impression Cytology with Immunofluorescence Staining

Impression cytology was performed on the first day and the 36th day by firmly attaching a nitrocellulose filter paper to the orbital conjunctiva of the participants. The procedure was performed on both eyes with the two filter papers prepared for immunofluorescence staining. The filter papers were fixed, washed, and subsequently incubated with anti-NLRP3 antibodies (1/500 dilution) (Cat# ab263899, Abcam, Cambridge, UK) at 4 °C overnight. Subsequently, they were exposed to an Alexa Fluor 488-conjugated secondary antibody. DAPI was used to stain the cell nuclei specifically. Visualization and photography of the results were performed using a Zeiss LSM510 META laser scanning microscope (Carl Zeiss, Oberkochen, Germany) equipped with an oil immersion objective.

### 2.9. Statistics

Statistical analysis was performed using GraphPad Prism (Version 10.4.1). The normality of continuous variables was assessed using the Shapiro–Wilk test, and the homogeneity of variance was verified using the Brown–Forsythe test. A parametric paired *t*-test was used for within-group comparisons, and ANOVA with Dunnett’s multiple comparison test was applied for comparisons across multiple groups. A *p* value < 0.05 was considered statistically significant. All values are expressed as mean ± SEM.

## 3. Results

### 3.1. Demographic Characteristics

A total of 68 DES participants were enrolled in the clinical trial. Thirty-eight participants were enrolled in the probiotic group and thirty in the placebo group (Figure 1).

The probiotic group consisted of 25 (65.8%) females and 13 (34.2%) males, averaging 42.3 ± 9.5 years. The placebo group enrolled 30 participants, consisting of 16 (53.3%) females and 14 (46.7%) males, with an average age of 44.4 ± 11.2 years (Table 2). Statistical analysis revealed no significant difference in gender distribution between the probiotic and placebo groups (*p* = 0.067). Similarly, normality in age was assessed using the Shapiro–Wilk test (*p* = 0.63), confirming that the data followed a normal distribution.

### 3.2. iHA318 Increases the Tear Volume

**Figure 2:**
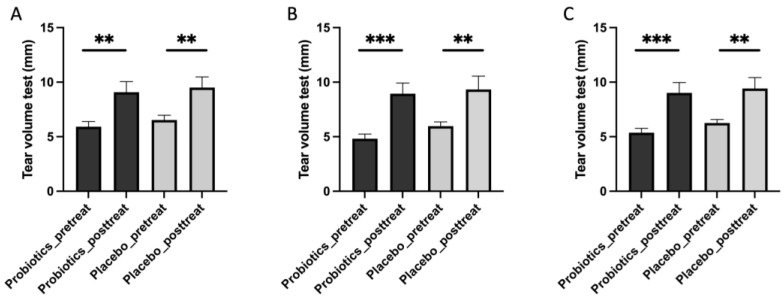
The tear volume before (pretreatment) and after (post-treatment) the administration of treatment in the probiotic and placebo groups in the right eyes (**A**), left eyes (**B**), and both eyes (**C**). Significant differences were represented as ** (*p* < 0.01), and *** (*p* < 0.001).

The tear volume plays a critical role in maintaining ocular surface health, with alterations often implicating a DES status. Reduced tear volume has been consistently associated with DES severity, highlighting the significance of tear volume assessment in diagnosing and managing this condition [27,28]. The tear volume measurements taken before and after treatment with probiotics or the placebo revealed significant changes in both groups (Figure 2) (Table 3). In the iHA318 probiotic group, there was a notable increase in tear volume for both the right eye (pretreatment: 5.92 ± 0.46 mm; post-treatment: 9.08 ± 0.96 mm; *p* value: 0.003) (Figure 2A) and the left eye (pretreatment: 4.82 ± 0.42 mm; post-treatment: 8.95 ± 0.95 mm; *p* value: 0.0001) (Figure 2B). The total average tear volume also showed a significant increase (pretreatment: 5.37 ± 0.39 mm; post-treatment: 9.01 ± 0.93 mm; *p* value: 0.0004) after the iHA318 probiotic treatment (Figure 2C). In the placebo group, there was an increasing trend in tear volume for both the right eye (pretreatment: 7.29 ± 0.87 mm; post-treatment: 9.50 ± 0.99 mm; *p* value: 0.004) (Figure 2A) and the left eye (pretreatment: 5.97 ± 0.39 mm; post-treatment: 9.33 ± 1.22 mm; *p* value: 0.006) (Figure 2B). The total average tear volume also exhibited an increase (pretreatment: 6.25 ± 0.33 mm; post-treatment: 9.42 ± 1.00 mm; *p* value: 0.002) following placebo administration (Figure 2C).

### 3.3. iHA318 Mitigates Tear Osmolarity

Tear osmolarity, serving as a critical indicator of ocular surface health, was employed to assess the impact of probiotic intervention on tear film [29]. The tear osmolarity data revealed distinct patterns between the probiotic and the placebo groups (Table 4). Participants in the probiotic group exhibited a significant decrease in tear osmolarity following treatment (pretreatment: 304.84 ± 2.42 mOsm/L, post-treatment: 299.55 ± 1.83 mOsm/L; *p* = 0.043), indicative of an improvement in tear film composition and hydration status, thus suggesting a favorable response after probiotic intervention. Conversely, the results of the placebo group did not demonstrate a significant change in tear osmolarity following treatment (pretreatment: 302.97 ± 2.90 mOsm/L, post-treatment: 305.17 ± 3.39 mOsm/L; *p* = 0.312), suggesting that the placebo treatment did not effectively modulate tear film stability or ocular surface hydration in individuals with DES.

### 3.4. iHA318 Prolongs the Tear Break-Up Time (TBUT)

**Figure 3:**
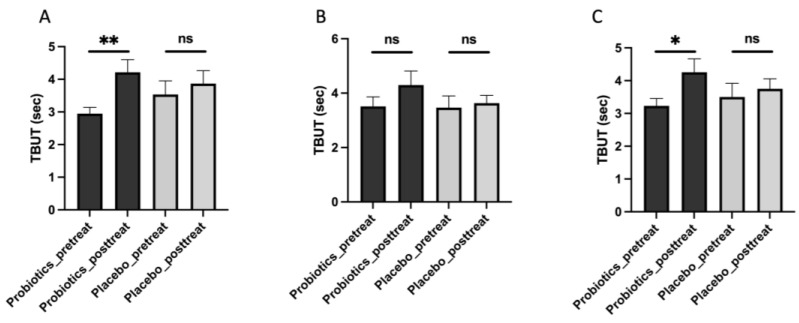
TBUT before (pretreatment) and after (post-treatment) the administration of treatment in the probiotic and placebo groups in the right eyes (**A**), left eyes (**B**), and both eyes (**C**). ns: not significant. Significant differences were represented as * (*p* < 0.05), and ** (*p* < 0.01).

To further assess the tear film stability and evaluate the efficacy of probiotic intervention for individuals with DES, TBUT testing was used in the present study [26]. An analysis of TBUT data revealed significant improvements in tear film stability following probiotic treatment (Table 5 and Figure 3). Specifically, participants in the probiotic group exhibited a significant increase in TBUT in the right eye (pretreatment: 2.95 ± 0.19 s, post-treatment: 4.22 ± 0.39 s; *p* = 0.023) and a notable trend towards improvement in the left eye (pretreatment: 3.51 ± 0.35 s, post-treatment: 4.30 ± 0.51 s; *p* = 0.105), with a significant increase in the total average TBUT (pretreatment: 3.23 ± 0.22 s, post-treatment: 4.26 ± 0.41 s; *p* = 0.015). Conversely, participants in the placebo group did not exhibit significant changes in TBUT for either eye or in the total average TBUT.

### 3.5. iHA318 Improves the Ocular-Related Serum Sialic Acid Level

To understand the effects of probiotic treatment in the clinical trial, we evaluated sialic acid (SA) serum levels as markers of ocular surface health [30]. Sialic acid plays a crucial role in maintaining mucin integrity in the tear film, influencing its viscosity and stability. Changes in SA levels can reflect alterations in tear film composition and quality [31]. In the probiotics group, a significant increase in SA levels (pretreatment: 2.52 ± 0.14, post-treatment: 2.93 ± 0.17, *p* value: 0.035) was found, indicating that it may improve tear film stability and enhanced mucin production (Table 6). Conversely, the placebo group showed no significant change in SA levels (pretreatment: 2.61 ± 0.14, post-treatment: 2.78 ± 0.17, *p* value: 0.228).

### 3.6. iHA318 Alleviates the Symptoms of Dry Eye Syndrome

**Figure 4:**
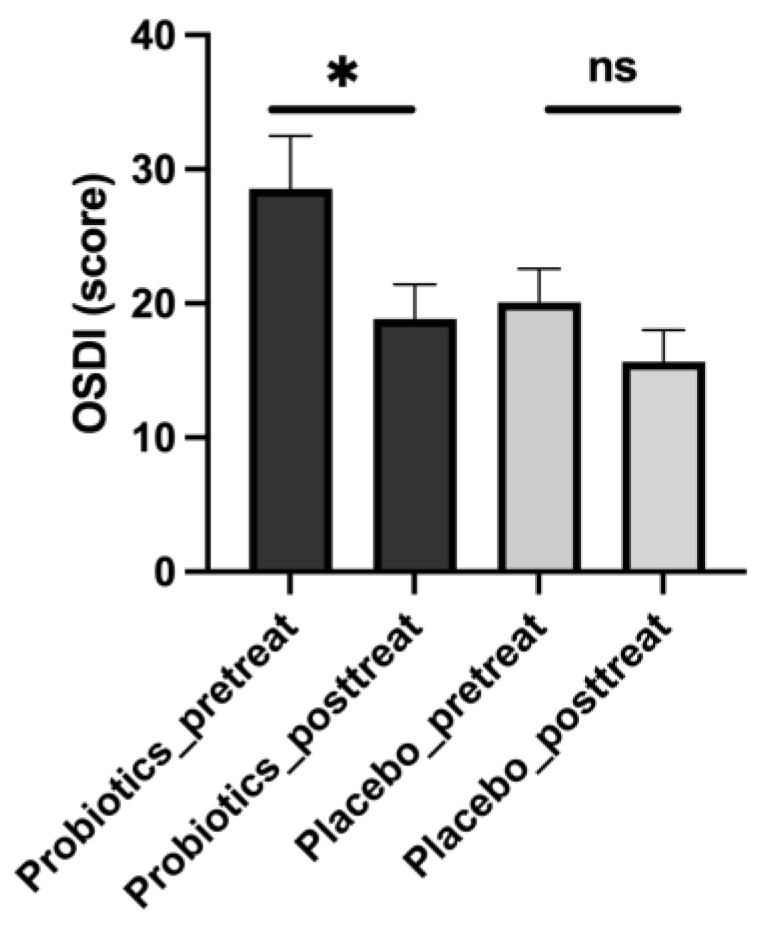
OSDI before (pretreat) and after (post-treat) administration of treatment in probiotic and placebo groups. ns: not significant. A significant difference was represented as * (*p* < 0.05).

To understand the impact of the probiotic treatment on ocular surface disease symptoms, we utilized the OSDI questionnaire as a standardized measure [25]. Table 7 and Figure 4 show that the results reveal significant improvements in ocular discomfort and visual disturbances following probiotic intervention. Specifically, the mean OSDI score decreased significantly from 28.56 ± 3.9 before treatment to 18.85 ± 2.58 after treatment (*p* value: 0.021), indicating a substantial reduction in the severity of ocular surface disease symptoms. Conversely, in the placebo group, no significant difference was observed between pretreatment and post-treatment OSDI scores (pretreatment: 20.08 ± 2.49, post-treatment: 15.66 ± 2.35, *p* value: 0.100).

### 3.7. iHA318 Suppresses NLRP3 Expression

**Figure 5:**
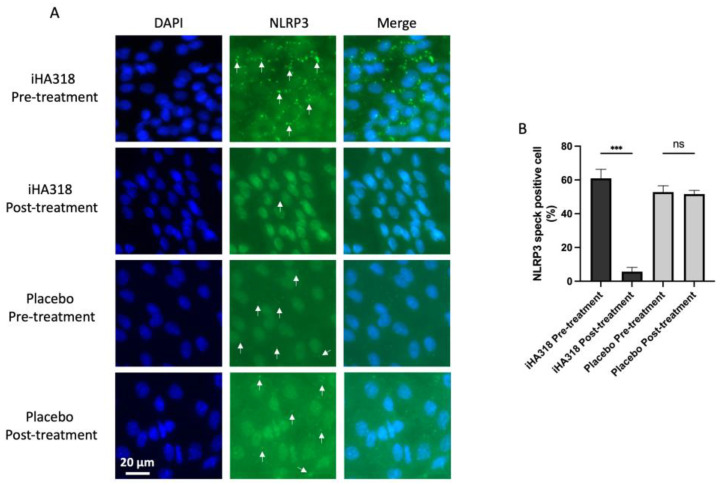
NLRP3 speck formation assay before (pretreatment) and after (post-treatment) treatment in probiotic and placebo groups. More NLRP3 specks were detected before iHA318 treatment, while no significant changes were observed in placebo group (**A**); quantitative analysis is shown in (**B**). White arrows indicate NLRP3 specks. ns: not significant. A significant difference was represented as *** (*p* < 0.001).

The NLRP3 inflammasome has been implicated in various ocular diseases, including DES, uveitis, and age-related macular degeneration, due to its role in mediating inflammatory responses within the eye [32,33,34]. Dysregulated NLRP3 signaling can contribute to chronic inflammation and tissue damage, highlighting its potential as a therapeutic target for mitigating ocular inflammation and preserving visual function. To investigate the impact of iHA318 probiotics on NLRP3 activation, we conducted an immunofluorescent microscopic assay to observe the formation of NLRP3 specks within conjunctival cells obtained from participants before and after treatment. The results show a significantly higher level of NLRP3 speck formation in the pretreatment group than the post-treatment group (Figure 5). In contrast to the treatment group, no significant change in NLRP3 expression was observed in the placebo group before and after the 35-day intervention, suggesting that the downregulation effect is specific to iHA318 and not attributable to time or placebo influence.

## 4. Discussion

DES presents a significant challenge in ocular health, characterized by insufficient tear production and chronic inflammation [35]. This study investigated the therapeutic potential of *Streptococcus thermophilus* iHA318, a probiotic strain, in alleviating dry eye symptoms and addressing chronic inflammatory responses. Through a double-blind design and the enrollment of 68 volunteers with DES, we aimed to comprehensively evaluate the efficacy of iHA318 probiotics through oral intake in managing this highly prevalent ocular condition.

Our analysis revealed several notable findings regarding the mitigating effects of *Streptococcus thermophilus* iHA318 on DES. Specifically, treatment with iHA318 significantly increased tear volume, improved tear osmolarity, prolonged the tear break-up time, and suppressed NLRP3 inflammasome activation. These results highlight the multifaceted effects of *Streptococcus thermophilus* iHA318 probiotics in addressing the complex pathophysiology of DES.

The clinical significance of the multifaceted effects is multi-fold. First, the increased tear volume secretion and TBUT would enhance tear film stability and ocular surface hydration following treatment with iHA318. These changes are critical for maintaining ocular surface health and alleviating the symptoms associated with DES. Second, normalizing tear osmolarity suggests improved tear film composition and reduced ocular surface irritation, further contributing to symptom relief in individuals with DES. Third, the suppression of NLRP3 inflammasome activation observed in this study may be attributed to immunomodulatory effects mediated by gut microbiota–immune system crosstalk [36,37,38]. It is well established that NLRP3 activation requires a two-signal mechanism: a priming signal via the NF-κB pathway and an activation signal from cellular stress or danger signals [39]. Oral probiotics, including *S. thermophilus* strains, have been shown to modulate the composition and function of intestinal microbiota, leading to systemic anti-inflammatory effects [23,40]. These effects include reduced NF-κB activation and altered cytokine profiles. Through these mechanisms, iHA318 may indirectly reduce ocular surface inflammation and downregulate NLRP3 expression at the conjunctival epithelium. This supports the emerging concept of a gut–immune–ocular axis, in which gut microbial modulation influences ocular immune homeostasis [38,41]. Further molecular studies are needed to verify the specific pathways involved, but our findings align with this systemic anti-inflammatory mechanism.

The increase in serum sialic acid level represents another mechanism to explain the mitigating effects of the oral intake of iHA318. Sialic acid is an important marker of ocular mucin, and its concentration in tear fluids is a marker of secreted mucins [42,43] and a target of novel therapeutic drugs for dry eye relief [44]. Sialic acid is a critical terminal glycan on mucins and plays a key role in maintaining the hydration and viscoelasticity of the tear film [45]. MUC5AC, the principal gel-forming mucin on the ocular surface, is rich in sialylated glycan chains, which contribute to tear film stability and ocular surface protection. Although this study observed a significant increase in serum SA levels after probiotic intervention, we did not measure SA concentrations in the tear film, which limits our ability to confirm a direct correlation. Nonetheless, several studies suggest that serum sialic acid levels may reflect mucin-associated barrier integrity and anti-inflammatory status systemically [46,47]. Thus, the observed increase in serum SA may indicate an enhanced mucin response at mucosal surfaces, including the ocular surface [43], resulting in more stable tear fluids on the ocular surface and potentially contributing to the mitigating effects against dry eye by iHA318. Future studies incorporating paired serum and tear fluid SA analysis are warranted to confirm this relationship and elucidate mechanistic pathways.

There are some limitations in the present study. Firstly, the relatively small sample size (n = 68) limits the statistical power and generalizability of our findings. While significant differences were detected in several key indicators, larger-scale studies are necessary to confirm these effects in diverse populations. Additionally, some placebo group improvements were observed, likely due to psychological expectations, trial engagement, and potential behavioral changes. Such responses are common in dry eye trials and may reflect the subjective nature of some endpoints (e.g., OSDI and TBUT). Despite this, multiple outcome measures (e.g., TV, TBUT, and OSDI) were assessed using paired *t*-tests without correction for Type I error inflation. Although the number of comparisons was limited and effect directions were consistent, this could introduce some false positive risk. Future studies with larger sample sizes and predefined primary endpoints should incorporate adjustments for multiple comparisons (e.g., Bonferroni corrections) to enhance statistical rigor. Additionally, while our study utilized a rigorous double-blind design, the potential placebo effects cannot be entirely discounted. It is possible that some participants in the placebo group experienced improvements in dry eye symptoms due to psychological or behavioral factors rather than the actual intervention. Future research should incorporate additional control measures, such as sham treatments or active placebos [48], to diminish the influence of placebo effects on study outcomes. Another limitation is that although prescription ocular therapy history (e.g., steroids or cyclosporine) was controlled for during recruitment, detailed documentation on the use of artificial tears was not collected. Since artificial tears may have mild lingering effects, future studies should consider implementing a washout period (e.g., 4 weeks) to better isolate the effect of the probiotic intervention. In addition, the etiological subtypes of DED (e.g., ocular surface-related vs. gut microbiota-related vs. autoimmune) were not subclassified in this trial. Although we excluded known systemic diseases, such as Sjögren’s syndrome, our cohort may still represent a heterogeneous population with variable pathogenesis. This heterogeneity may influence treatment response and limits the mechanistic specificity of our findings. Future trials could incorporate microbiome profiling, inflammatory markers, or stratified inclusion based on the DED subtype to more precisely validate the therapeutic relevance of the gut–eye axis. Additionally, although TBUT was measured using a standardized protocol, only one reading was obtained per eye. We acknowledge that clinical guidelines recommend averaging three consecutive TBUT measurements to reduce variability and enhance reliability. This limitation should be addressed in future studies to ensure a more rigorous assessment of tear film stability. Furthermore, although our study demonstrated the suppression of NLRP3 inflammasome activation by iHA318, further investigation is needed to fully elucidate the underlying mechanisms. Specifically, the effects of iH318 to potentially inhibit inflammatory pathways [49], ocular surface microbiota [50], and immune modulation [51] in individuals with DES demand extensive investigation. In addition, this study did not quantify the sialic acid contents in the tear fluid to elucidate the direct contribution of increased serum sialic acid contents. Eventually, clinical trials evaluating the long-term safety and efficacy of iHA318 in larger patient populations are warranted to validate its therapeutic potential and establish an evidence-based adjunctive treatment strategy for DES.

Despite these limitations, our study contributes valuable evidence supporting the potential of *Streptococcus thermophilus* iHA318 as a therapeutic intervention for individuals with DES.

## 5. Conclusions

The present results suggest that the oral administration of *Streptococcus thermophilus* iHA318 represents a promising therapeutic option for individuals with DES, offering evidence-based efficacy for ocular health and inflammation management. The multifaceted benefits potentiate iHA318 as a valuable adjunctive therapy for individuals with DES, highlighting the potential of probiotic intervention in managing ocular surface diseases.

## Figures and Tables

**Table 1:** The components of the administrated capsules.

	Main Ingredient	Dosage Regiment
Probiotic capsule ^†^	*Streptococcus thermophilus* iHA318 and microcrystalline cellulose (excipient)	5 × 10^9^ CFU/capsule, 1 capsule/day for 35 days
Placebo capsule	Microcrystalline cellulose	1 capsule/day for 35 days

^†^ Capsule ingredients: hydroxypropyl methylcellulose, H_2_O, carrageenan, and KCl.

**Table 2:** Demographics between participants with DES assigned to probiotic group and placebo group.

Variable	Probiotic Group	Placebo Group	Total	*p* Value ^†^
n (%)	38 (55.9)	30 (44.1)	68 (100)	
Gender, n (%)				
Female	25 (65.8)	16 (53.3)	41 (60.3)	0.067
Male	13 (34.2)	14 (46.7)	27 (39.7)	
Age (years)	42.3 ± 9.5	44.4 ± 11.2	42.9 ± 10.2	0.630

^†^ The *p* value for gender was assessed using a chi-square test, while the *p* value for age group was determined using a Shapiro–Wilk test.

**Table 3:** Tear volume changes between probiotic and placebo groups.

Variable	Probiotic (n = 38)		Placebo (n = 30)	
	TV (mm)	*p* Value ^†^	TV (mm)	*p* Value ^†^
OD (right eye)				
Pretreatment	5.92 ± 0.46		7.29 ± 0.87	0.916
Post-treatment	9.08 ± 0.96 **	0.003	9.50 ± 0.99 **	0.004
OS (left eye)				
Pretreatment	4.82 ± 0.42		5.97 ± 0.39	0.267
Post-treatment	8.95 ± 0.95 ***	0.0001	9.33 ± 1.22 **	0.006
Both eyes				
Pretreatment	5.37 ± 0.39		6.25 ± 0.33	0.437
Post-treatment	9.01 ± 0.93 ***	0.0004	9.42 ± 1.00 **	0.002

^†^ The *p* value for the tear volume measurement was determined using a *t*-test, and the homogeneity of variance was analyzed using the Brown–Forsythe test, representing significant differences as ** (*p* < 0.01), and *** (*p* < 0.001). Abbreviations: TV (tear volume), OD (oculus dextrus—the right eye), OS (oculus sinister—the left eye). The quantitative values are presented as the mean ± SEM.

**Table 4:** Tear osmolarity changes between probiotic and placebo groups.

Variable	Probiotic (n = 38)		Placebo (n = 30)	
	Osmolarity (mOsm/L)	*p* Value ^†^	Osmolarity (mOsm/L)	*p* Value ^†^
Pretreatment	304.84 ± 2.42		302.97 ± 2.90	0.996
Post-treatment	299.55 ± 1.83 *	0.043	305.17 ± 3.39	0.312

^†^ The *p* value for tear osmolarity measurement was determined using a *t*-test, and the homogeneity of variance was analyzed using the Brown–Forsythe test, representing significant differences as * (*p* < 0.05). The quantitative values are presented as the mean ± SEM.

**Table 5:** TBUT changes between probiotic and placebo groups.

Variable	Probiotics (n = 38 ^‡^)		Placebo (n = 30)	
	TBUT (second)	*p* Value ^†^	TBUT (second)	*p* Value ^†^
OD (right eye)				
Pretreatment	2.95 ± 0.19		3.53 ± 0.42	0.744
Post-treatment	4.22 ± 0.39 **	0.023	3.87 ± 0.40	0.283
OS (left eye)				
Pretreatment	3.51 ± 0.35		3.47 ± 0.43	0.831
Post-treatment	4.30 ± 0.51	0.105	3.63 ± 0.29	0.374
Total (average)				
Pretreatment	3.23 ± 0.22		3.50 ± 0.42	0.993
Post-treatment	4.26 ± 0.41 *	0.015	3.75 ± 0.31	0.316

^†^ The *p* value for TBUT measurement was determined using a *t*-test, and the homogeneity of variance was analyzed using the Brown–Forsythe test, representing significant differences as * (*p* < 0.05), and ** (*p* < 0.01). Abbreviations: TBUT (tear break-up time), OD (oculus dextrus—the right eye), OS (oculus sinister—the left eye). The quantitative values are presented as the mean ± SEM. ^‡^ One participant’s data were excluded due to the outlier value caused by incomplete blinking during the TBUT test.

**Table 6:** Serum sialic acid (SA) level changes in the probiotic group and the placebo group.

Variable	Probiotics (n = 38) ^‡^		Placebo (n = 30)	
SA (mmol/L)		*p* Value ^†^		*p* Value ^†^
Pretreatment	2.52 ± 0.14		2.61 ± 0.14	0.998
Post-treatment	2.93 ± 0.17 *	0.035	2.78 ± 0.17	0.228

^†^ The *p* value for sialic acid measurement was determined using a *t*-test, and the homogeneity of variance was analyzed using the Brown–Forsythe test, representing a significant difference as * (*p* < 0.05). Abbreviation: SA (sialic acid). The quantitative values are presented as the mean ± SEM. ^‡^ One data point was excluded due to missing data.

**Table 7:** OSDI score changes between probiotic and placebo groups.

Variable	Probiotics (n = 38)		Placebo (n = 30)	
	OSDI Score	*p* Value ^†^	OSDI Score	*p* Value ^†^
Pretreatment	28.56 ± 3.9		20.08 ± 2.49	0.358
Post-treatment	18.85 ± 2.58 *	0.021	15.66 ± 2.35	0.100

^†^ The *p* value for the OSDI measurement was determined using a *t*-test, and the homogeneity of variance was analyzed using the Brown–Forsythe test, representing significant differences as * (*p* < 0.05). The quantitative values are presented as the mean ± SEM.

## Data Availability

The associated pre-processed raw data are available and can be shared with interested parties upon reasonable request. Please contact the corresponding author David Pei-Cheng Lin (email: pcl@csmu.edu.tw) for more information.

## Abbreviations

The following abbreviations are used in this manuscript:

TV	Tear Volume
TBUT	Tear Break-Up Time
Osmo	Osmolarity
SA	Sialic Acid
OSDI	Ocular Surface Disease Index
DES	Dry Eye Syndrome
DED	Dry Eye Disease
